# H-ferritin-nanocaged gadolinium nanoparticles for ultra-sensitive MR molecular imaging

**DOI:** 10.7150/thno.93856

**Published:** 2024-02-24

**Authors:** Jianlin Zhang, Chang Yuan, Lingfei Kong, Feiyan Zhu, Wanzhong Yuan, Junying Zhang, Juanji Hong, Fang Deng, Qi Chen, Chen Chen, Tao Wang, Zhentao Zuo, Minmin Liang

**Affiliations:** 1 Experimental Center of Advanced Materials, School of Materials Science & Engineering, Beijing Institute of Technology, Beijing, 100081, China.; 2 Center of Basic Medical Research, Institute of Medical Innovation and Research, Peking University Third Hospital, Beijing, China.; 3 National Laboratory of Biomacromolecules, Institute of Biophysics, Chinese Academy of Sciences, Beijing, China.; 4 Department of Neurosurgery, Peking University Third Hospital, Beijing, 100191, China.; 5 State Key Laboratory of Brain and Cognitive Science, Institute of Biophysics, Chinese Academy of Sciences, Beijing, 100101, China.

**Keywords:** magnetic resonance imaging, human H-ferritin, contrast agent, relaxivity value, molecular imaging

## Abstract

**Rationale:** Magnetic resonance imaging (MRI) is a powerful diagnostic technology by providing high-resolution imaging. Although MRI is sufficiently valued in its resolving morphology, it has poor sensitivity for tracking biomarkers. Therefore, contrast agents are often used to improve MRI diagnostic sensitivity. However, the clinically used Gd chelates are limited in improving MRI sensitivity owing to their low relaxivity. The objective of this study is to develop a novel contrast agent to achieve a highly sensitive tracking of biomarkers *in vivo*.

**Methods:** A Gd-based nanoprobe composed of a gadolinium nanoparticle encapsulated within a human H-ferritin nanocage (Gd-HFn) has been developed. The specificity and sensitivity of Gd-HFn were evaluated *in vivo* in tumor-bearing mice and apolipoprotein E-deficient mice (Apoe^-/-^) by MRI.

**Results:** The Gd-HFn probe shows extremely high relaxivity values (*r*_1_ = 549 s^-1^mM^-1^, *r*_2_ = 1555 s^-1^mM^-1^ under a 1.5-T magnetic field; and *r*_1_ = 428 s^-1^mM^-1^ and *r*_2_ = 1286 s^-1^mM^-1^ under a 3.0-T magnetic field), which is 175-fold higher than that of the clinically standard Dotarem (Gd-DOTA,* r*_1_ =3.13 s^-1^mM^-1^) under a 1.5-T magnetic field, and 150-fold higher under a 3.0-T magnetic field. Owing to the substantially enhanced relaxivity values, Gd-HFn achieved a highly sensitive tracking for the tumor targeting receptor of TfR1 and enabled the *in vivo* MRI visualization of tumors approaching the angiogenic switch.

**Conclusions:** The developed Gd-HFn contrast agent makes MRI a more powerful tool by simultaneously providing functional and morphological imaging information, which paves the way for a new perspective in molecular imaging.

## Introduction

Magnetic resonance imaging (MRI) is emerging as a powerful noninvasive clinical diagnostic technology that provides high-resolution anatomic images of living organisms on a microscopic and macroscopic scale 1,2. However, the diagnostic sensitivity of MRI is relatively low and thus 40-50% of clinical MRI examinations have used contrast agents to enhance the detection sensitivity. Signal intensity in MRI is related to the proton relaxation rates of H_2_O in the surroundings tissues and can be enhanced by the administration of paramagnetic contrast agents prior to imaging. These administrated paramagnetic agents improve MRI anatomical images on the basis of their ability to increase the relaxation rates of nearby water proton spins. Gd chelates are by far the most commonly employed commercial MRI contrast agents in the clinic because of their strong paramagnetic properties 3,4. However, their limited relaxivities, lack of selectivity, and difficulty in further functionalization prevent their further involvement in improving MRI sensitivity 5,6. In addition, due to the toxicity of Gd ion (LD50 = 0.2 mmol kg ^-1^ in mice), the induced high risks of nephrogenic systemic fibrosis for patients with renal dysfunction and Gd deposition disease also severely impede the further clinical applications of Gd chelates 7,8. Therefore, it is critical to develop high-performance and safe Gd-based contrast agents by stabilizing toxic Gd ion while keeping the hydration number high and the water exchange rate optimal.

Ferritin is a spherical iron storage protein shell composed of 24 self-assembled subunits of two types, H-chain ferritin (heavy chain, 21 kDa, HFn) and L-chain ferritin (light chain, 19 kDa, LFn). Ferritin self-assembles naturally into a hollow protein cage with a 12 nm of outer diameter and an 8 nm diameter of interior hollow cavity 9. The interior cavity of ferritin protein cage has been extensively employed as a natural template for preparing highly monodisperse and water-soluble metallic nanoparticles (NPs) 10-13. We have previously reported that human HFn could specifically accumulate into tumors and atherosclerotic plaques via transferrin receptor 1 (TfR1)-mediated specific targeting followed by rapid internalization 14-19. Herein, we synthesized a highly monodisperse Gd core within the interior cavity of HFn nanocage and achieved a sensitive MRI tracking of TfR1 with a remarkable enhanced relaxivity of water protons. The protein entrapment of Gd nanoparticle within HFn nanocage allows a substantively increased dipolar interactions of Gd with H_2_O molecules nearby and significantly enhanced proton relaxation within the interior confined space of HFn protein nanocage. In addition, we further demonstrated the feasibility of simultaneously acquiring functional parameters using this highly sensitive contrast agent, in addition to high-resolution anatomic MRI information.

So far, several Gd chelate complexes, including Gd-DTPA 20, Gd-Me(2)DO_2_A 21, and GdHPDO_3_A 22,23 have been loaded or conjugated to ferritin for MRI study. However, due to the unchanged fundamental coordination chemistry of the chelated Gd ions, only less than 10-fold enhancement in relaxivities has been achieved and the main parameters that affect their efficacy as a proton relaxation agent have rarely been changed. Very recently, Cigler et al. reported that the released Gd^3+^ ions from Gd chelates administrated intravenously bind to the iron oxyhydroxide core inside ferritin capsules under physiological conditions 24. Yang et al. recently synthesized a Gd^3+^ compound and packaged it into ferritin nanocages for MRI imaging and multimodal therapy of tumors simultaneously 25. These two works suggest a new strategy to develop Gd-based contrast agents by empolying ferritin platform. In this study, we show a bioengineered HFn-nanocaged Gd nanocore that achieved simultaneous MRI functional and morphological imaging, which paves the way for a new perspective in MRI molecular imaging.

## Results

### Development and characterization of Gd-HFn

**Figure 1:**
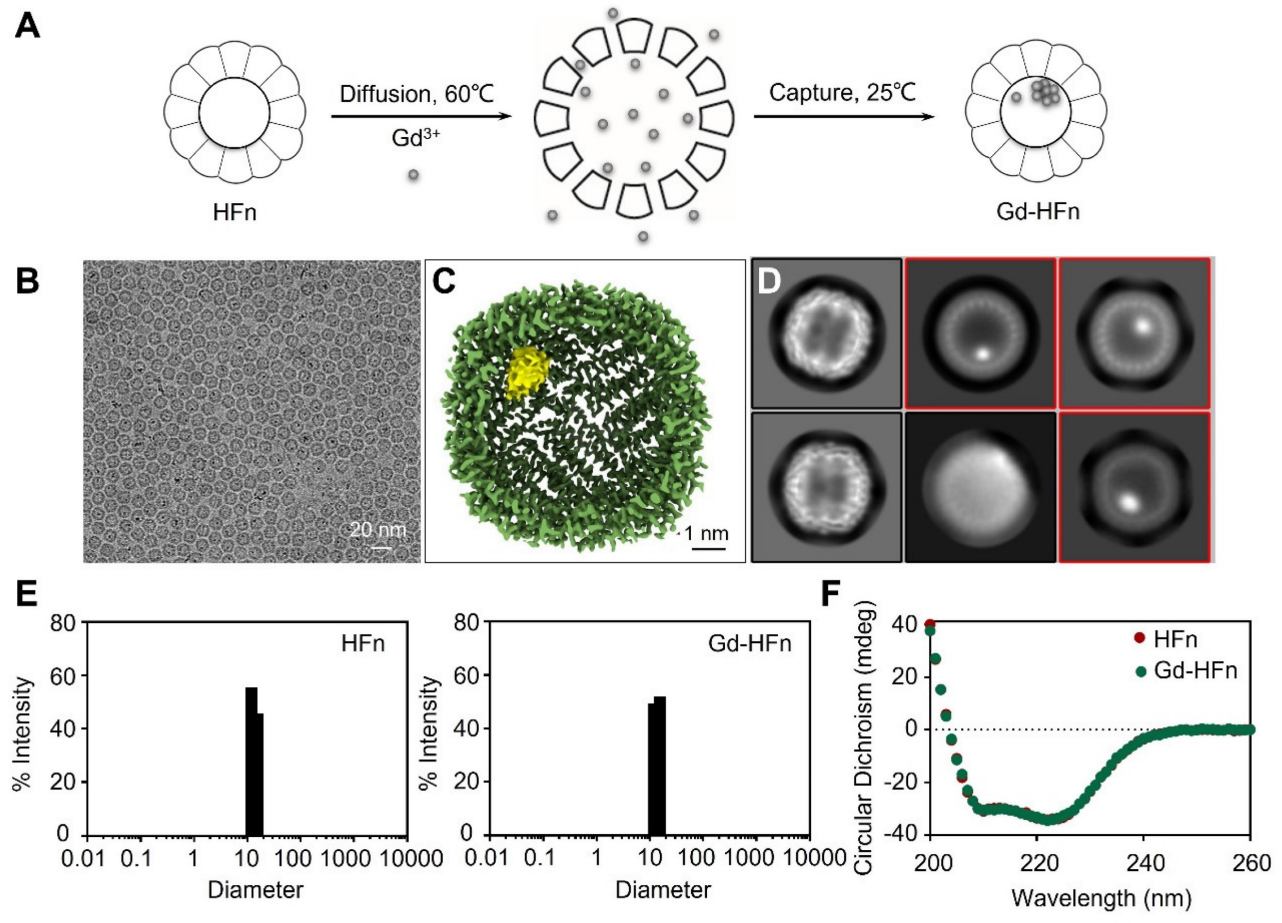
** Preparation and characterization of Gd-HFn. (A)** Schematic illustration of the preparation process of Gd-HFn NPs. **(B)** Cryo-EM images of the as-synthesized Gd-HFn. **(C)** Cryo-EM density map of the as-synthesized Gd-HFn determined at 3.6 Å resolution. HFn is coloured in Green, Gd are coloured in yellow. Scale bar represents 1 nm. **(D)** 2D class averages of Gd-HFn. Selected 2D classes are boxed in red. **(E)** DLS analysis of HFn nanocages (left) and Gd-HFn (right). **(F)** Circular dichroism spectra of HFn and Gd-HFn.

Recombinant human HFn nanocages were expressed and purified from *E. coli*, as descried previously 14. Gd-HFn NPs were prepared by loading Gd^3+^ into the cavities of HFn nanocages through the ion channel at the threefold axis of HFn nanocage, followed by the formation of Gd nanoparticle within the nucleation site (Figure 1A). After purification, the obtained Gd-HFn NPs were thoroughly characterized. Cryo-electron microscopy (cryo-EM) images first showed that the formation of the uniformly and monodispersed Gd NPs with an average diameter of ~1.0 nm that are clearly encapsulated within the HFn protein nanocages (Figure 1B-D). Inductively coupled plasma optical emission spectrometry (ICP-OES) quantitatively determined the amount of encapsulated Gd to be 37± 2 gadolinium per HFn nanocage (Figure S1). Dynamic light scattering (DLS) analysis further confirmed that the bioengineered Gd-HFn NPs are highly monodispersed with identical diameters before and after forming Gd NPs within HFn protein nanocages (Figure 1E). Size exclusion chromatography (SEC) characterization showed HFn and Gd-HFn NPs were eluted at the same volume, indicating that HFn nanocages are assembled intactly after encapsulating Gd^3+^ cations (Figure S2). Moreover, the CD spectra result of Gd-HFn NPs was identical to that of HFn nanocages (Figure 1F), further confirming that Gd^3+^ loading did not significantly perturb the secondary structure of HFn protein nanocages, showing that HFn nanocages refold into their native state upon the loading process. The stability of the prepared Gd-HFn was next evaluated by incubating Gd-HFn with 10% mouse serum at 37 °C, and monitored for Gd release using ICP-OES. Less than 10% Gd release was detected within 60 h of incubation (Figure S3), suggesting that the Gd-HFn NPs is sufficiently stable to retain Gd during transit through the systemic circulation. Further cell viability, necrosis, and apoptosis assays show no obvious cell toxicity even at a very high concentration of Gd-HFn (containing 2 mM Gd), which could cause significant toxicity while 2 mM of free Gd-DOTA was added (Figure S4), confirming the biosafety of Gd-HFn.

### MR contrast properties of Gd-HFn

**Figure 2:**
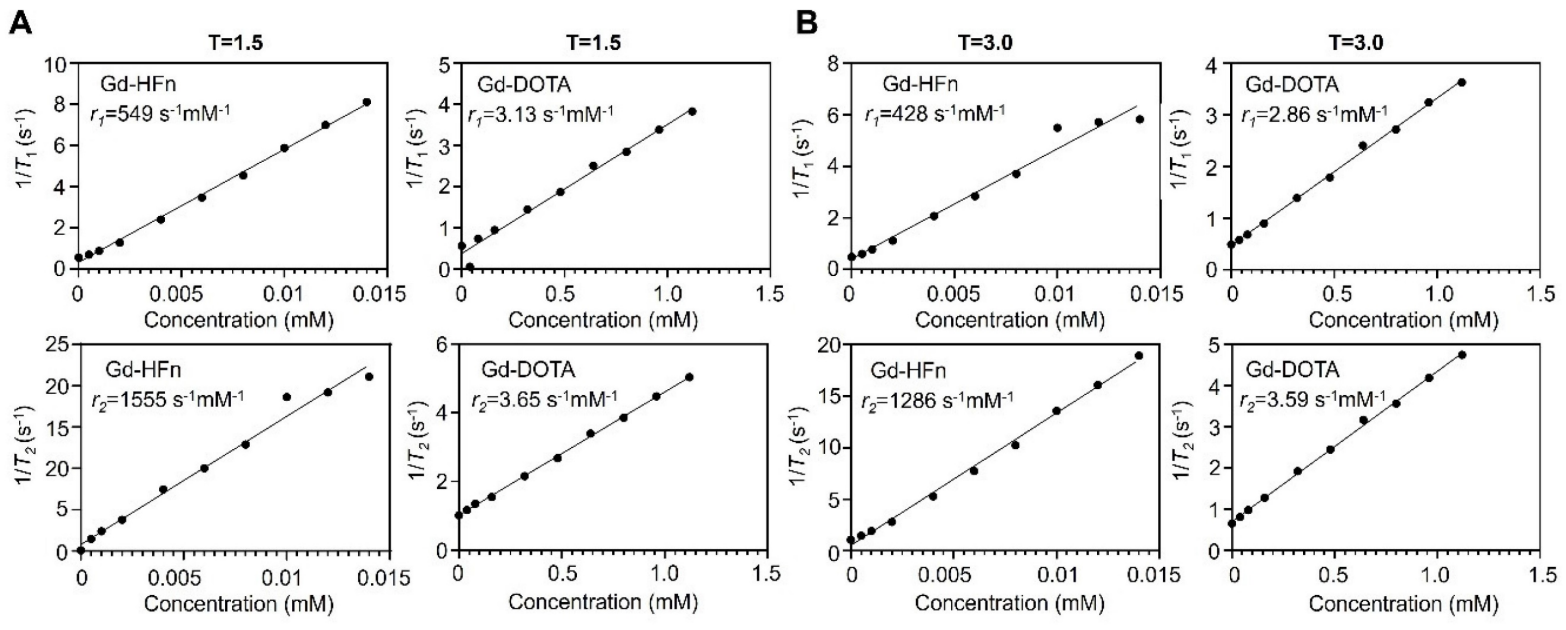
** MR contrast properties of Gd-HFn. (A)** 1/*T*_1_ and 1/*T*_2_ relaxation time plot of Gd-HFn and Gd-DOTA at different concentrations under a 1.5-T magnetic field. **(B)** 1/*T*_1_ and 1/*T*_2_ relaxation time plot of Gd-HFn and Gd-DOTA at different concentrations under a 3.0-T magnetic field.

The MR contrast properties of the biosynthesized Gd-HFn NPs were evaluated *in vitro* at room temperature. The longitudinal (*T_1_*) and transverse (*T_2_*) relaxation times were measured respectively at different concentrations of Gd-HFn, and the relaxivities were determined to be *r*_1_ = 549 s^-1^mM^-1^, *r*_2_ = 1555 s^-1^mM^-1^ per Gd under a 1.5-T magnetic field, and *r*_1_ = 428 s^-1^mM^-1^, *r*_2_ = 1286 s^-1^mM^-1^ per Gd under a 3.0-T magnetic field (Figure 2), surpassing any previously reported relaxivity values to the best of our knowledge 26. Gd-HFn showed about 175-fold higher *r*_1_ relaxivity than that of the clinically standard Gd-based *T_1_* contrast agent Dotarem (Gd-DOTA, 3.13 s^-1^mM^-1^) under a 1.5-T magnetic field, and about 150-fold higher under a 3.0-T magnetic field (Figure 2A, B). The measured *r*_2_/*r*_1_ ratio of Gd-HFn were 2.8 and 3.0, respectively under the magnetic field strength of 1.5-T and 3.0-T, which is lower than 5, demonstrating that Gd-HFn should be a high-performance *T*_1_ contrast agent at 1.5-T and 3-T magnetic field 27. The extremely high relaxivity values are likely due to the nanoscale confinement effect from HFn protein nanocage that contributes to their high MR contrast effect, which influences the paramagnetic behavior of the encapsulated Gd ions 28-30.

### MR imaging of small tumors

**Figure 3:**
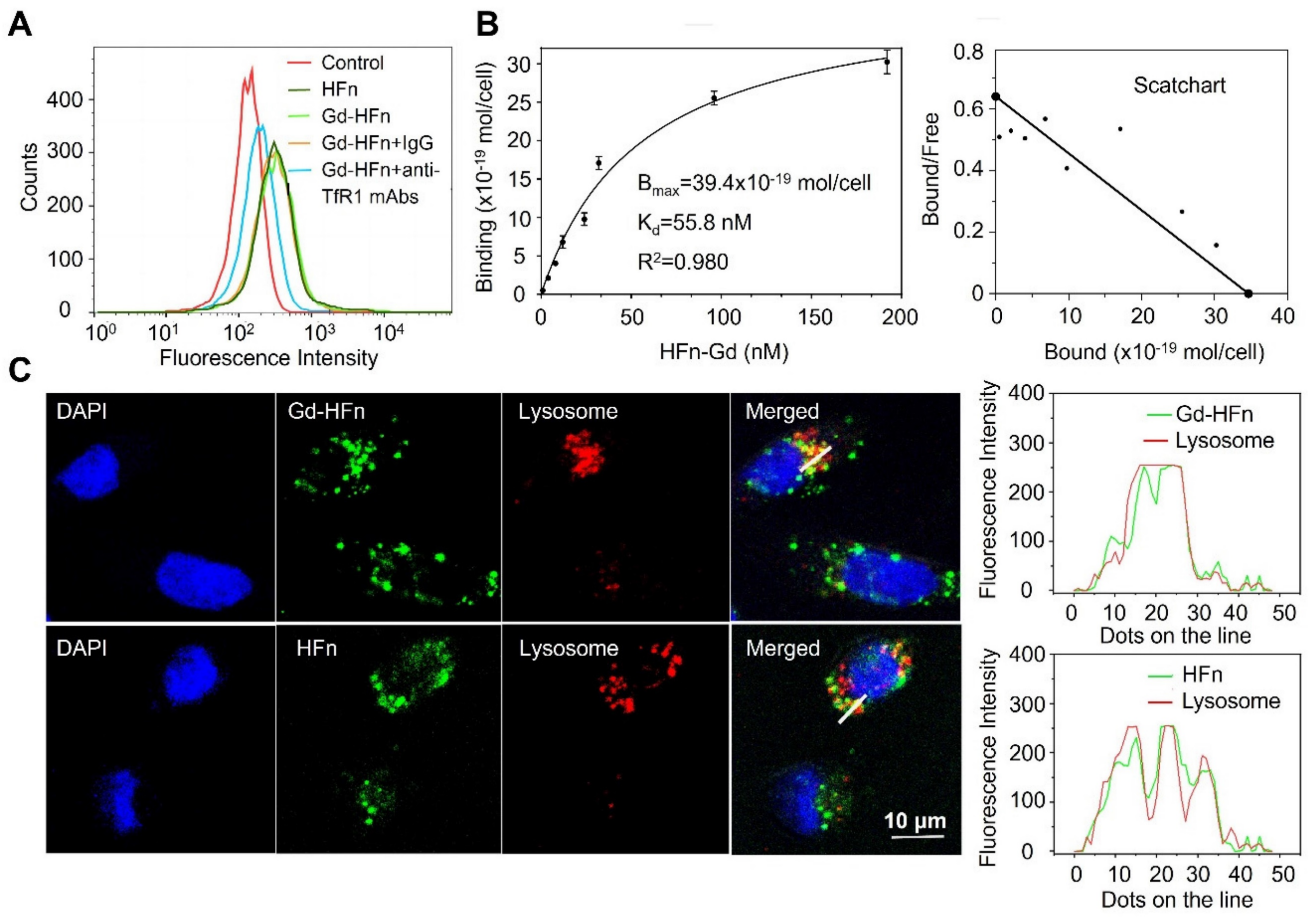
** Characterization of Gd-HFn binding to tumor cells. (A)** Flow cytometric analysis of the specific binding of Gd-HFn to MDA-MB-231 breast cancer cells. **(B)** FITC-conjugated Gd-HFn bound to MDA-MB-231 cancer cells with a *K*_d_ value of 55.8 nM and a total binding capacity *B*_max_ of 39.4 × 10^-19^ mol/cell. (n=3 independent measurements, error bars represent mean ± s.d.). **(C)** Confocal images of the intracellular location of HFn and Gd-HFn in the lysosomes of MDA-MB-231 tumor cells. The right panels show the fluorescent signal profiles of the lysosome and the HFn or Gd-HFn along the lines drawn in the Merged pictures.

We have previously reported that HFn protein can selectively bind to human tumors and significantly accumulate into tumor cells via TfR1-mediated specific targeting and the subsequent robust internalization 14,15. The prepared HFn nanocages thus should be able to deliver the confined Gd NPs into tumor cells and perform Gd-based tumor MR imaging. We next investigated the specific uptake of Gd-HFn NPs into tumor cells. As shown in Figure 3A, Gd-HFn NPs substantially bound to MDA-MB-231 human breast cancer cells and the binding was significantly blocked by adding an excess of anti-TfR1 monoclonal antibody (mAb) as determined by flow cytometric analysis, confirming a specific binding of Gd-HFn to TfR1 on MDA-MB-231 tumor cells. The further saturation binding curve and Scatchard analysis demonstrate that the K_d_ value for Gd-HFn binding to MDA-MB-231 tumor cells is 55.8 nM and the total binding capacity B_max_ is 39.4 × 10^-19^ mol/cell (Figure 3B), showing that Gd-HFn has a high binding affinity and capacity for TfR1-positive tumor cells. The next confocal images further show that the specific targeting of Gd-HFn NPs to MDA-MB-231 tumor cells leads to the further internalization and the subsequent trafficking of Gd-HFn into lysosomes (Figure 3C). We next quantitatively investigated the specific cell uptake of Gd-HFn in TfR1-positive MDA-MB-231 and TfR1-negtive MX-1 cells. We incubated MDA-MB-231 and MX-1 cells with 40 μg/mL of Gd-HFn for 8 h at 37 °C with constant motion. After the incubated cells were washed with PBS, the Gd uptake was determined using ICP measurements after digesting the cells. The results show that MDA-MB-231 internalized about 80 pmol of Gd per 10^6^ cells (Figure S5). In contrast, a slight uptake was observed at TfR1-negtive MX-1 cells, indicating the TfR1-mediated specific selectivity.

**Figure 4:**
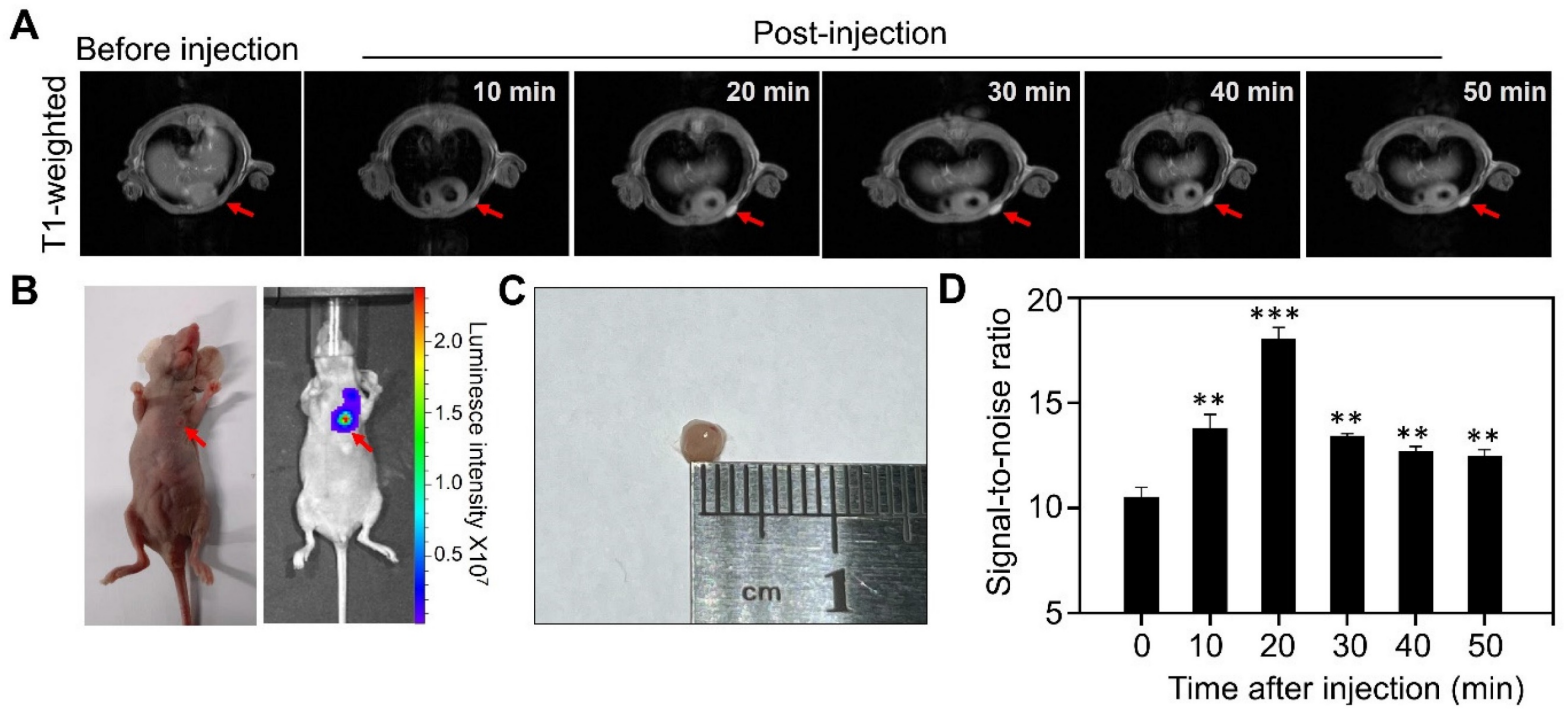
** MR imaging of Micro tumors in living mice with Gd-HFn. (A)** Representative MR imaging of the cross section of the MDA-MB-231 tumors implanted in mice before and after the injection of Gd-HFn. Arrows mark the tumor location. **(B)** Optical imaging of MDA-MB-231tumors implanted mice. **(C)** Image of the single tumor excised from the mice after the imaging. **(D)** Quantitative analysis of the signals in the tumor (marked by the arrows) showing a significantly increase as compared to before the injection. (n=5 independent measurements, error bars represent mean ± s.d., unpaired Student's t-test, **P < 0.01, ***P < 0.001 versus before injection).

We next evaluated the MR performance of Gd-HFn *in vivo* by T1-weighted MR imaging on MDA-MB-231 tumor-bearing mice with an MRI scanner. We employed an orthotopic breast tumor model in which we implanted Luciferase-transfected human MDA-MB-231 breast cells (Luciferase^+^ MDA-MB-231) in the mammary fat pad of female mice. When the tumor diameters reached about 2 mm, we injected tumor-bearing mice (n=5) through tail vein with Gd-HFn and performed MR imaging on each mouse before injection and at 10 min, 20 min, 30 min, 40 min and 50 min after injection, respectively (Figure 4). The images from after the injection demonstrated clear visualization of the tumors (Figure 4A). In parallel to the MR imaging of the mice, we also acquired optical images using the fluorescence signals from Luciferase^+^ MDA-MB-231 that were co-registered with the MRI image (Figure 4B), showing good colocalization between the MR and optical imaging. Image of the single tumor excised from the mice after the imaging showed the tumors for MR imaging were about 2 mm (Figure 4C). Quantification of the signal in the region of interest (ROI) of the tumors showed a significantly increase in the MR imaging as compared to before the injection of Gd-HFn (Figure 4D). The MR imaging signal-to-noise ratio increased from 10.4±0.5 before injection to maximum 18.0±0.6 at 20 min after injection. The contrast enhancement of Gd-HFn-based imaging became more remarkable when used for imaging large tumors (Figure S6). The *in vivo* imaging results showed that the high MR sensitivity of Gd-HFn enables the MR visualization of tumors as small as ~2 mm in diameter, which is approaching the angiogenic switch (1~2 mm) 31-33, the transition from diffusion-limited nutrition to neovascularization. The results indicate a meaningful tumor imaging of small numbers of malignant cells because tumor growth typically shows Gompertzian kinetics 34 with a lag phase starting from the single cell stage, and a log phase heralded by angiogenesis that promote tumor further progression and metastasis 35. Gd-HFn thus shows the potential of imaging the smallest possible number of tumor cells before the angiogenic switch.

### MR imaging of atherosclerotic plaques

**Figure 5:**
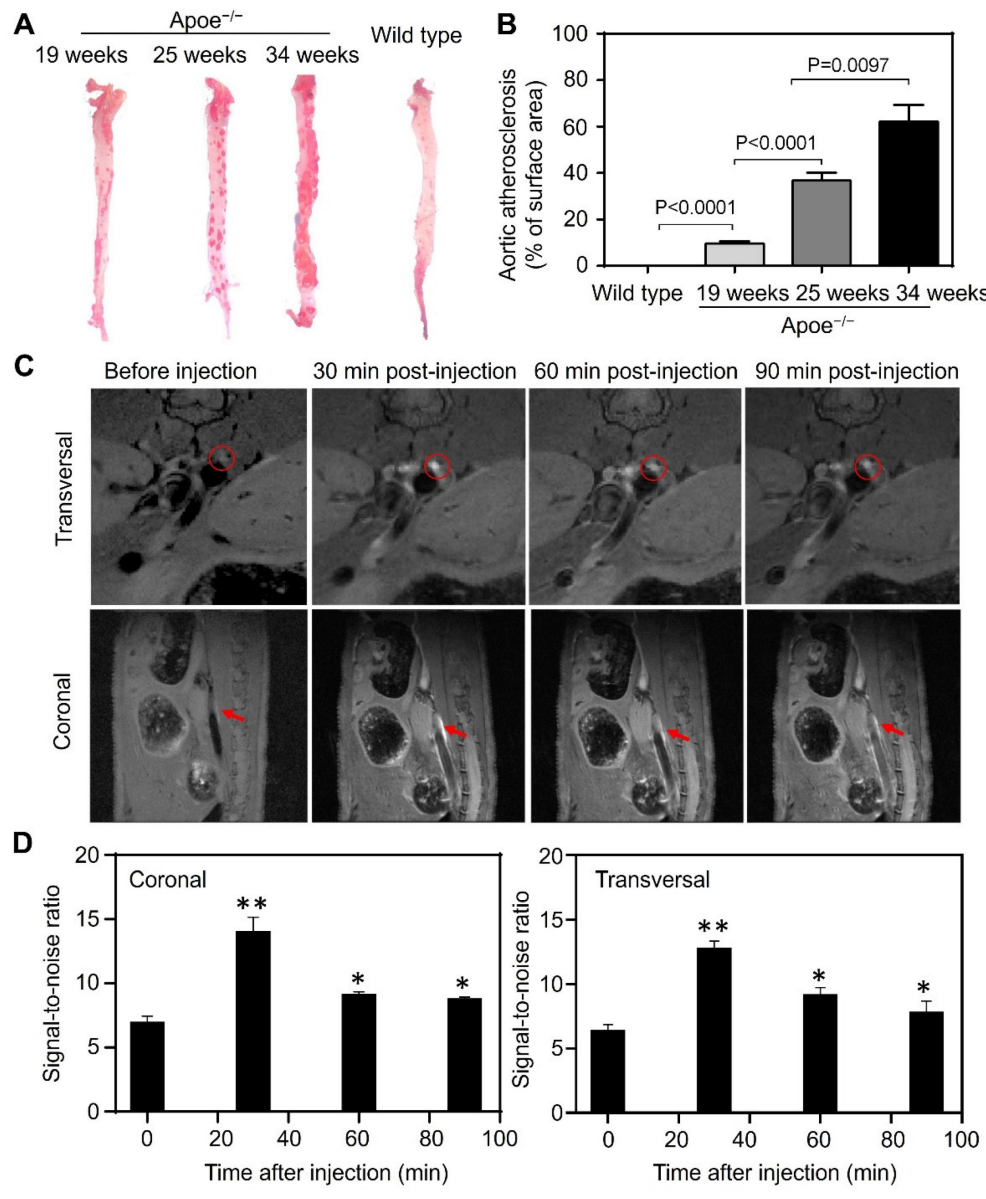
** MR imaging of atherosclerotic plaques in living mice with Gd-HFn. (A)** Representative *ex vivo* images of Oil Red O staining of aortas from Apoe^-/-^ and control mice. **(B)** Quantitative analysis of the atherosclerotic plaque area of the entire aorta. (n=5 independent measurements, error bars represent mean ± s.d., unpaired Student's t-test). **(C)** Representative MR imaging of Apoe^-/-^ mice carrying atherosclerotic plaques before and after the injection of Gd-HFn. Red circles and arrows indicate atherosclerotic plaques. **(D)** Quantitative analysis of the signals in the atherosclerotic plaques (marked by the red circles or arrows) showing a significantly increase as compared to before the injection. (n=5 independent measurements, error bars represent mean ± s.d., unpaired Student's t-test, *P < 0.05, **P < 0.01 versus before injection).

Progressive inflammation drives atherosclerotic plaque formation, progression and rupture and are the compelling diagnostic targets for identifying high-risk plaques 36-38. We have previously reported TfR1 receptors are overexpressed on a subset of the macrophage population in high-risk plaques 16. Moreover, increased expression of HFn have been found in the macrophages after infiltrated into human high-risk plaques 39,40. The developed Gd-HFn contrast agents thus can be employed for MR imaging of plaque vulnerability. We first evaluated the feasibility of MR imaging of atherosclerotic plagues by using Gd-HFn in apolipoprotein E-deficient mice (Apoe^-/-^) fed with a high-fat diet for 19, 25, and 34 weeks, respectively, to develop varying degrees of atherosclerosis. Wild-type C57/BL6 mice were employed as disease controls. The progressive development of atherosclerosis in Apoe^-/-^ mice was evaluated by Oil Red O staining of the aortas excised from Apoe^-/-^ mice (Figure 5A, B). We performed MR imaging on atherosclerotic and control mice before injection and at 10 min, 20 min, 30 min, 40 min and 50 min post-intravenous injection of Gd-HFn (Figure 5C). The images from after the injection showed clear visualization of atherosclerotic plaques in the aortas of atherosclerotic mice both in the transversal and coronal planes. In contrast, only indistinct images of atherosclerotic plaques in the aortas were observed from before the injection. The MRI signal-to-noise ratio of atherosclerotic plaques for transversal and coronal planes increased respectively from 11.1±0.7 and 11.9±0.2 before intravenous injection to maximum 16.8±1.1 and 18.4±1.1 at 30 min after injection of Gd-HFn NPs (Figure 5D). The results demonstrated the feasibility of MR imaging atherosclerotic plaques with Gd-HFn.

We next evaluated the* in vivo* toxicity of Gd-HFn in healthy BALB/c mice by detecting the loss of mice body weight after intravenous injection at the same dose of Gd-HFn (0.016 mmol Gd/kg body weight). Gd-HFn at 0.016 mmol Gd/kg body weight was well tolerated with no significant weight loss as compared with PBS controls during imaging procedures (Figure S7). We next assessed the biosafety of Gd-HFn NPs by pathological analysis of the main organs (including heart, liver, spleen, lung and kidney) from healthy mice after intravenous injection of Gd-HFn at the MR imaging dose (Figure S8). No abnormal organ pathological abnormality was found in the main organs of mice treated with Gd-HFn, showing the safety of Gd-HFn imaging at the tested doses.

We next evaluated the pharmacokinetics and biodistribution of Gd-HFn in healthy BALB/c mice. The elimination blood half-life (*t_(_*_1/2)β_) of Gd-HFn was found to be 126 ± 7.0 min, whereas the blood half-life of Gd-DOTA was 24 ± 3.1 min (Figure S9A). The significantly longer blood half-life indicates improved drug retention in the systemic circulation and facilitates time-dependent Gd-HFn accumulation in disease targets. In addition, the background Gd concentration in major healthy organs decreased over the period of evaluation (Figure S9B), indicating a rapid clearance of Gd-HFn from the whole body, which thus provides a clear background for imaging targets. Moreover, the next blood chemistry data also exhibited no significant differences as compared with the PBS-treated control group (Table S1), further confirming the biosafety of Gd-HFn imaging at the tested doses. The results demonstrate that by encapsulating Gd into HFn protein nanocages significantly improves the biosafety of heavy metal Gd and thus increases the absolute concentration of Gd into the target lesion.

## Discussion

We designed and tested a unique MRI contrast agent that represents an advance for MRI technique and may have a potential impact for the following reasons: (1) High relaxivity: Gd-HFn is, to our knowledge, the MRI contrast agent with the extremely high relaxivity values (*r*_1_ = 549 s^-1^mM^-1^ and *r*_2_ = 1555 s^-1^mM^-1^ under a 1.5-T magnetic field, and *r*_1_ = 428 s^-1^mM^-1^ and *r*_2_ = 1286 s^-1^mM^-1^ under a 3.0-T magnetic field), which is about 175-fold higher *r*_1_ relaxivity than that of the clinically standard Dotarem (Gd-DOTA, 3.13 s^-1^mM^-1^) under a 1.5-T magnetic field, and about 150-fold higher* r*_1_ relaxivity under a 3.0-T magnetic field. The *r*_1_ and *r*_2_ values are much better when compared to most Gd-based contrast agents described in the literature (Table S2). (2) Specificity: Human HFn specifically binds to TfR1, which has been found overexpressed in many pathological conditions such as tumors 14,15 and high-risk atherosclerotic plaques 16,18,41. With the significantly improved relaxivity, Gd-HFn thus could achieve a sensitive biomarker tracking of TfR1 and enabled the *in vivo* visualization of tumors and high-risk atherosclerotic plaques via TfR1-mediated specific targeting. (3) Biocompatibility: The employed HFn nanocage is an iron storage protein that naturally exists in the human body and thus exhibits excellent biocompatibility when used *in vivo*. Moreover, HFn protein nanocages require no further functionalization with extra targeting ligands that might induce immune responses against the bioengineered Gd-HFn NPs. In addition, the toxic Gd ions are encapsulated within HFn nanocages and sufficiently stable passing through the systemic circulation. Nevertheless, the following systematic evaluation is still need to evaluate the biosafety of Gd-HFn for *in vivo* MR imaging. (4) Simplicity and easy scaling-up: HFn is expressed and produced from *E. coli* at high yield and the Gd ion payload process within HFn nanocage is simple and efficient. Importantly, the intrinsic target binding property of Gd-HFn with no need of extra surface modification enables a large-scale of reproducibility and further facilitates future clinical translation.

In summary, we report here an effective Gd-loaded HFn contrast agent for MR imaging. The bioengineered Gd-HFn showed good stability, safety, and high-sensitive tracking of the biomarker TfR1. This Gd-HFn contrast agent exhibited extremely high relaxivity values and enabled the *in vivo* MRI visualization of plaque vulnerability and ultra-small tumors approaching the angiogenic switch (~2 mm). We believe clinical MRI would demonstrate superior imaging characteristics by employing the developed Gd-HFn contrast agent.

## Materials and Methods

### Materials

Anhydrous calcium chloride, anhydrous sodium carbonate, and ethyl alcohol were purchased from Sinopharm Chemical Reagent Co. Ltd (Shanghai, China). Poly(acrylic acid) (PAA, Mw ≈ 1800) was purchased from Aldrich. Gadolinium nitrate (cat. no. 05660), Fluorescein isothiocyanate isomer I (FITC, cat. no. F7250), RPMI-1640 medium (cat. no. 11875093) and fetal bovine serum (FBS, cat. no. A5669501) was from Sigma. Gadolinium trinitrate (cat. no. R053801) was from Shaghai Rhawn Chemical Reagent Co., Ltd (Shanghai, China). All reagents were used as received without further purification. The commercially used Dotarem (Gd-DOTA) was produced by Guangdong Consun Pharmaceutical Group, China. BCA Protein Assay Kit (cat. no. A55864) was from Thermo Scientific.

### Biosynthesis of Gd-HFn

Recombinant human HFn was expressed in *E. coli* and purified as descried previously 17. The Gd-HFn NPs were prepared as follows. Briefly, gadolinium trinitrate was diluted to 120 μM and then added dropwise at a speed of 100 μL/min into 1 mL of stirring HFn solution (1 mg/mL; in 20 mM Tris-HCl, pH 8.0 containing 0.1 M NaCl) which was kept at 60 ℃ in a water bath. The dropping time was set as 30 min, 60 min, 90 min, 120 min, 150 min, 180 min, 210 min, 240 min. After the dropping was finished, the solution was stirred for another 120 min. The reaction vessel was then removed from the water bath and cooled naturally to the room temperature. The obtained solution was then dialyzed against the buffer of 20 mM Tris-HCl (pH 8.0) containing 0.1 M NaCl overnight to remove the free Gd cations.

### Characterization

Transmission electron microscopy (TEM) (JEM-1400 Flash, Nippon Electronics, Japan) of HFn and Gd-HFn NPs was performed on TF20 high-resolution EM (FEI). Size-exclusion chromatography (SEC) (GE Healthcare, UK) analysis was performed on a HiLoad 16/600 Superdex 200 prep grade size exclusion chromate-graphy column (GE Healthcare, UK) connected to an AKTA avant 150 system (GE Healthcare, UK). Dynamic light scattering (DLS) (Wyatt Technology, USA) analysis of HFn and Gd-HFn NPs was performed on a DynaPro Titan system DLS instrument at 25 ℃. Circular Dichroism (CD) (Applied Photophysics, UK) spectra HFn and Gd-HFn nano-particles was performed on an Applied Photophysics Chirascan Plus spectrometer at 25 ℃.

### Gadolinium quantification

Gadolinium quantification was measured using inductively coupled plasma optical emission spectrometry (ICP-OES, Thermo Scientific, model no. iCAP6300). Briefly, 0.2 mL of HFn-Gd NPs (0.5 mg/mL) were first digested in nitric acid at 150 ℃ in a metal bath thermostat for 10 min to dissolve HFn protein and the gadolinium content inside the cavity, and then 2% nitric acid was added to make the final reaction volume up to 10 mL. ICP-OES was settled as below: the radio frequency power is 1.0 KW, the carrier gas is Argon, the plasma flow is 15 L/min, the auxiliary gas flow is 1.5 L/min, the nebulizer gas flow is 0.75 L/min, the detector mode is axial mode, and the calibration type is linear. The duration of the process is 15 min. The digested HFn-Gd solution was analyzed for Gd using the ICP-OES. The standard curve of Gd was established using gadolinium nitrate with concentrations of 0.0, 0.1, 0.3, 0.5, 1.0, and 2.0 mg/mL. The Gd content was finally calculated according to the established standard curve.

### Labeling of HFn and Gd-HFn

Fluorescein isothiocyanate isomer I (FITC) was dissolved in DMSO to 80 μM, and then added to HFn or Gd-HFn solution (4 μM in the buffer of 100 mM carbonate pH 9.0). The mixture was incubated at 4 ℃ overnight, and then purified with a PD MiniTrap G25 column (GE Healthcare) to remove the free FITC. The concentration of conjugated FITC was determined by measuring its optical density at 492 nm, the concentration of the HFn protein was determined using a BCA Protein Assay Kit.

### Cell binding assay

MDA-MB-231 human breast cancer cells were from American Type Culture Collection (ATCC, NM-R03) and were cultured in RPMI-1640 medium containing 10% FBS. Flow cytometry was used to evaluate the specific binding of HFn or Gd-HFn NPs to MDA-MB-231 cells. In brief, 100 μL of cell suspension (1×10^7^ cells/mL) was incubated with 10 μg/mL FITC-HFn or FITC-Gd-HFn nano-particles on ice for 30 min. Mouse anti-human TfR1 antibody (10 μg/mL) or mouse IgG (10 μg/mL) was added in the mixture when needed. After rinsed in cold PBS for three times, the cells were analyzed immediately using a FACSCalibur flow cytometry system (Becton Dickinson).

### Cellular uptake and distribution

The cellular uptake and distribution of HFn and Gd-HFn NPs were studied using a confocal laser scanning microscope (Olympus). In brief, MDA-MB-231 cells were incubated with FITC-HFn or FITC-Gd-HFn NPs (10 μg/mL) for 2 h at 37℃ in cell incubator. Then the cells were fixed with cold 4% paraformaldehyde for 30 min. 0.5% Triton X-100 was used to permeabilized the cells. After incubated with 5% goat serum, the cells were exposed to the primary anti-Lamp1 antibody (1:200; clone H4A3; Invitrogen), and then incubated with Alexa flour 647R-conjugated goat anti-mouse secondary antibodies (1:500, Invitrogen). Finally, the cells were stained with 4′,6-diamidino-2-phenylindole (DAPI) and observed under a fluorescence microscope.

### *In vitro* MRI study

MRI experiments were performed on 1.5-T (Siemens Esensa) and 3.0-T clinical MRI scanner (Siemens Prisma) equipped with a microsurface coil. Spin-Lattice and Spin-Spin relaxation times (*T_1_* and *T_2_*) were measured for different concentrations of HFn-Gd and Gd-DOTA in PBS at room temperature using the following parameters: field of view (FOV) = 22 cm × 22 cm, matrix = 320 × 320, repetition time (TR) = 5000 ms, echo time (TE) = 10, 20, 30, 40, 50, 60, 70, 80, 90, 100..., 320 ms (*T_2_*), TR = 4000 ms, and TE = 7.8 ms (*T_1_*). The longitudinal (*r_1_*) and transverse (*r_2_*) relaxivies were calculated from *r_2_* = (1/*T_i_*-1/*T_0_*)/*c*, where c is the concentration of HFn-Gd or Gd-DOTA in mM, *T_i_* is the relaxation time at concentration c, *T_0_* is the relaxation time of PBS, and *i* = 1 and 2 for *T_1_* and *T_2_*, respectively**.**

### *In vivo* MRI study

All animal experiments were performed in compliance with the guidelines for the care and use of laboratory animals and were approved by the biomedical research ethics committee of the Beijing Institute of Technology under the accreditation number V20200118. Female Balb/c nude mice (5-6 weeks, Charles River) were administered by injecting 0.2 mL of MDA-MB-231 cell suspension (1×10^7^ cells/mL) at their second pair of mammary fat pads to establish the in situ mammary cancer model. When the tumor grew to 2-mm, MRI was performed on the tumor-bearing mice in the small-animal systems of the Bruker Biospec MRI scanner. 1.5% isoflurane in pure oxygen was used to anesthetize the mice. Gd-HFn NPs were injected through the tail vein of the mice at a dose of 0.016 mmol Gd/kg animal body weight. A RARE sequence was continuously used for T1-weighted imaging for 1.5 h post-injection. The parameters for the mice were as follows: FOV = 4.0 cm × 4.0 cm, matrix = 256 × 256, TR = 550 ms, TE = 6.5 ms, and 8 slices at 1 mm thicknesses without gap. MR images were acquired by using Paravision 360 preclinical MRI software.

ApoE^-/-^ mice (5-6 weeks, Charles River) were developed atherosclerosis by high-fat feeding (21% (wt/wt) fat; 0.15% (wt/wt) cholesterol) for 25 weeks. Oil red O staining was used to conform the existence of atherosclerotic plaques on the aorta. In brief, the aorta was cut from the mouse body and then was split. After being fixed by 10% formalin for 10 min, the split aorta was stained with oil red O for 30 min at 37 °C. The atherosclerotic plaques were taken pictures using a digital camera. After conforming the development of atherosclerosis, the mice in the same group were taken to MRI. The administrations of the mice and the MRI conditions are the same as the described above for the MRI of the tumor-bearing mice. A RARE sequence was continuously used for T1-weighted imaging for 1.5 h post-injection. The parameters for the mice were as follows: FOV = 2.56 cm × 2.56 cm, matrix = 256 × 256, TR = 920 ms, TE = 6.5 ms, and 8 slices at 0.5 mm thicknesses with 0.5 mm gap. MR images were acquired by using Paravision 360 preclinical MRI software.

### Biodistribution and histopathological examinations

For biodistribution study, healthy BALB/c mice were administered by injecting Gd-HFn through the tail vein at a dose of 0.016 mmol Gd/kg animal body weight. At 1, 3, 9 and 24 h post-injection, the mice were sacrificed. The major tissues (including heart, liver, spleen, lung, kidney and brain) were collected, weighted and homogenized with an addition of 1:10 (wt/vol) acidified isopropanol in a tissue homogenizer on ice and extracted overnight at -20 ℃. All homogenate samples were then centrifuged at 4 °C for 10 min at 18,000 × g. The supernatant was removed and the remaining Gd cations in each sample was measured using ICP-OES. The results were presented as percentage of injected dose (%ID) per gram of tissue. Values are expressed as means ± s.d. for a group of five mice.

For histopathological examinations, healthy BALB/c mice were administered by injecting Gd-HFn NPs through the tail vein at a dose of 0.016 mmol Gd/kg animal body weight. At 2 weeks post-injection, the mice were killed. The major tissues (including heart, liver, spleen, lung, and kidney) were collected and fixed in 4% paraformaldehyde. A routine paraffin-embedded hematoxylin and eosin (H&E) histopathology staining procedure was performed for histopathological examinations.

### Pharmacokinetics study

To determine the pharmacokinetic behavior, healthy BALB/c mice were administered by injecting Gd-HFn (0.016 mmol Gd/kg body weight) or Gd-DOTA (0.016 mmol Gd/kg body weight) through the tail vein (n = 5 for each group). At various times after injection, blood samples were collected from the tail vein and the plasma was separated and analyzed for Gd concentration using ICP-OES.

### Statistical analysis

All statistical analyses were performed with GraphPad Prism 5.01 (GraphPad Software Inc.) software. Results were expressed as mean ±s.d. Unpaired Student's t tests were performed to evaluate the significance of differences between groups. A value of P < 0.05 was considered statistically difference (*P<0.05, **P < 0.01, ***P < 0.001).

## Supplementary Material

Supplementary figures and tables.
