# Supplementary material for: H-ferritin-nanocaged gadolinium nanoparticles for ultra-sensitive MR molecular imaging
Source: Theranostics. 2024 Feb 24;14(5):1956–65. doi: 10.7150/thno.93856 (PMC10945347; doi:10.7150/thno.93856)
Supplement: Supplementary file 1 — Supplementary figures and tables. [file thnov14p1956s1.pdf]

Supporting Information for

H-ferritin–nanocaged gadolinium nanocores for ultra-sensitive MR molecular imaging

Jianlin Zhang[1,2#], Chang Yuan[1#], Lingfei Kong[3#], Feiyan Zhu[1], Wanzhong Yuan[4], Junying Zhang[1], Juanji Hong[1], Tao Wang[4*], Zhentao Zuo[5*], Minmin Liang[1*]

[1]Experimental Center of Advanced Materials, School of Materials Science & Engineering, Beijing Institute of Technology, Beijing, 100081, China.

[2]Center of Basic Medical Research, Institute of Medical Innovation and Research, Peking University Third Hospital, Beijing, China.

[3]National Laboratory of Biomacromolecules, Institute of Biophysics, Chinese Academy of Sciences, Beijing, China.

*Correspondence to:

Tao Wang, MD. Email: wangtao@bjmu.edu.cn

Zhentao Zuo, PhD. Email: zuozt@ibp.ac.cn

Minmin Liang, PhD. Email: mmliang@bit.edu.cn

#These three authors contributed equally.

Contents

1. Supporting Figures ………………………………………………………...... S2-7

2. Supporting Tables……………………………………………….………………S8-12

1. Supporting Figures

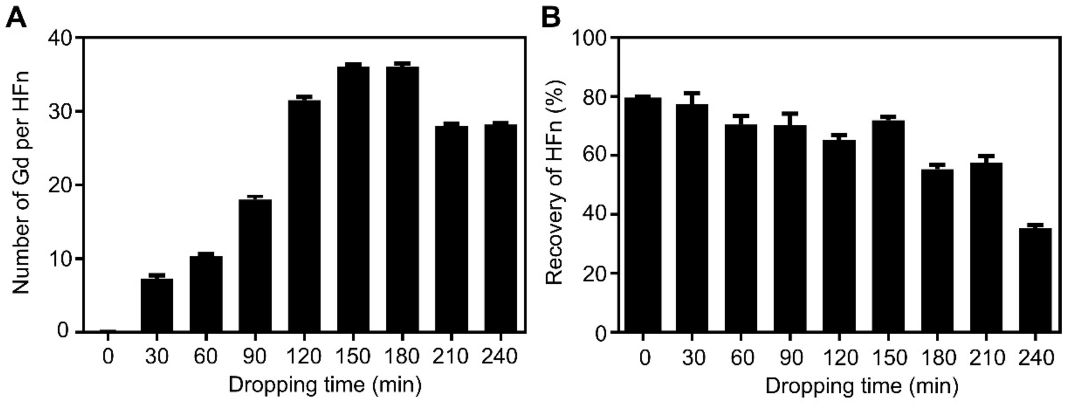

Figure S1. (A) Number of loaded Gd per HFn nanocage depends on the Gd dropping time. **(B)** HFn recovery yield (%) depends on the Gd dropping time. The encapsulated Gd was quantified using ICP-OES, and the HFn concentration was determined by using BCA protein assay kit.

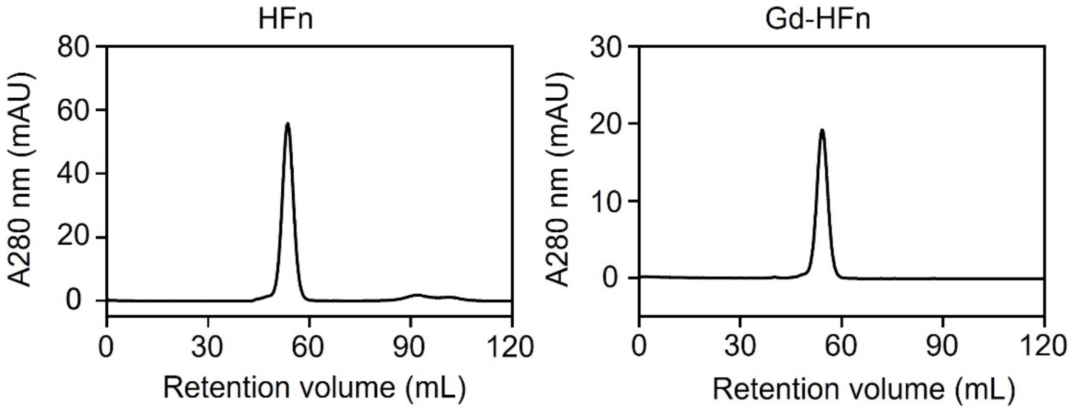

Figure S2. Representative SEC analysis of the HFn and Gd-HFn NPs.

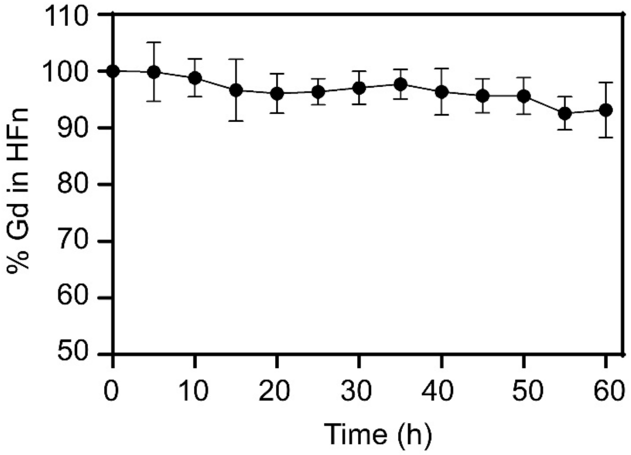

Figure S3. Stability of Gd-HFn NPs in 10% mouse serum at 37 ºC over 60 h of incubation (n = 3, bars represent means ± s.d.)

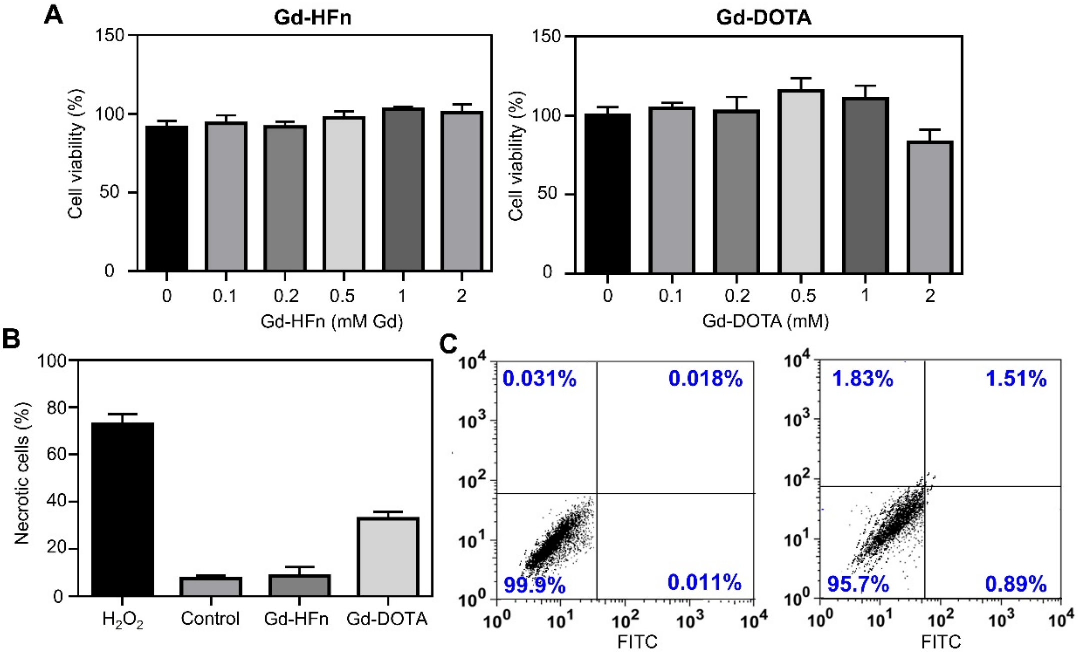

Figure S4. (A) Cell viability of MDA-MB-231 cells after treatment with Gd-HFn or Gd-DOTA for 24 h over a dose range of 0-2 mM Gd. CCK8 assay was used for cell viability assessment. (n = 5, bars represent means ± s.d.) **(B)** The percentage of necrotic cells measured by quantification of LDH release in the cell medium after incubation in the presence or absence (Negative control) of Gd-HFn or Gd-DOTA (2 mM Gd). Hydrogen peroxide (H_2O_2) was used as positive control. Results are expressed as percentage of dead cells with respect to the control. (n = 5, bars represent means ± s.d.) **(C)** Cell apoptosis analysis was performed using Annexin V-FITC assay using flow cytometry. Left, MDA-MB-231 cells untreated. Right, MDA-MB-231 cells treated with Gd-HFn for at concentration of 2 mM Gd.

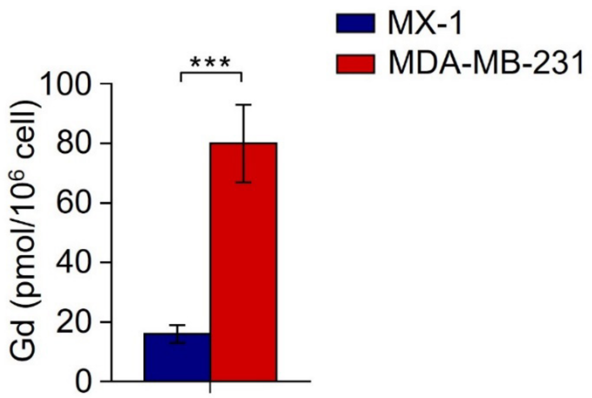

Figure S5. Cellular uptake of Gd-HFn measured by ICP-OES (n = 5, mean ± SD, unpaired Student's t-test, ***P < 0.001).

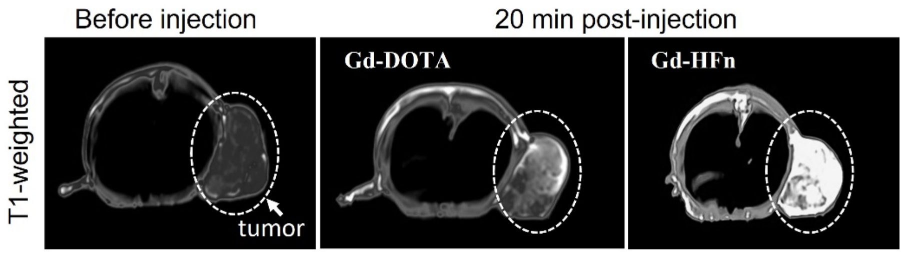

Figure S6. MR imaging of large tumors with Gd-DOTA or Gd-HFn in living mice.

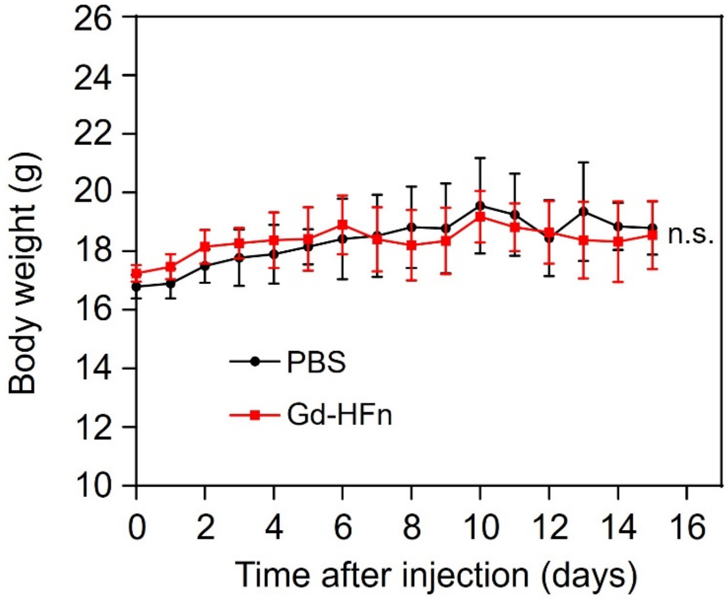

Figure S7. Toxicity evaluation of Gd-HFn *in vivo*. Healthy female BALB/c mice were administered intravenously on day 0 of Gd-HFn (0.016 mmol Gd/kg body weight) or PBS (n = 6 per group, bars represent means ± s.d.). n.s., not significant.

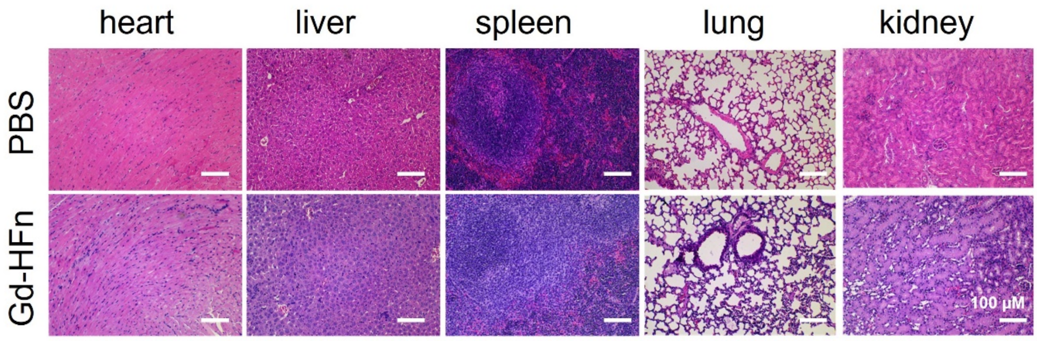

Figure S8. Representative images of organ histology examination from mice administered with PBS or Gd-HFn at a dose of 0.016 mmol Gd/kg animal body weight. Tissue slices were stained with hematoxylin and eosin (H&E). No noticeable abnormality was found in the heart, liver, spleen, lung, and kidney.

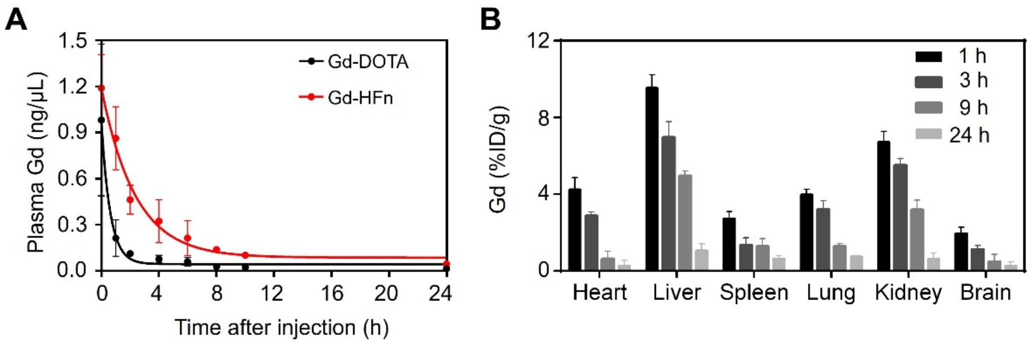

Figure S9. Total body clearance of Gd-HFn. (A) Plasma concentrations of Gd as a function of time after injection. Five healthy mice were intravenously injected with Gd-DOTA or Gd-HFn at a dose of 0.016 mmol Gd/kg animal body weight. Plasma samples at different time points were drawn and the plasma concentrations of Gd were measured by ICP-OES. (n=5 independent measurements, error bars represent mean ± s.d.). **(B)** Biodistribution of Gd-HFn. Tissue samples at different time points were drawn and the tissue concentrations of Gd were measured by ICP-OES. Data are presented as percentage of injected dose (%ID) per gram of tissue. Values are expressed as means ± s.d. for a group of five animals.

Table S1. Blood routine parameters of healthy mice treated with PBS, Gd-DOTA or Gd-HFn.

Blood routine parameters	PBS (n=5)		Gd-DOTA (n=5)		Gd-HFn (n=5)		Normal arrange
	1 Week	2 Weeks	1 Week	2 Weeks	1 Week	2 Weeks	
**WBC**	3.96±0.76	8.94±0.96	4.55±0.64	8.78±0.70	4.34±0.87	8.82±1.27	0.80-10.60
**NEU**	0.42±0.18	1.08±0.17	0.68±0.13	1.04±0.08	0.83±0.24	1.27±0.24	0.23-3.60
**LYM**	3.41±0.75	7.52±0.89	3.71±0.61	7.09±0.63	3.28±0.67	6.77±1.23	0.60-8.90
**MON**	0.06±0.01	0.25±0.05	0.09±0.01	0.36±0.04	0.10±0.02	0.31±0.07	0.04-1.40
**EOS**	0.05±0.02	0.24±0.09	0.06±0.01	0.23±0.04	0.11±0.06	0.21±0.07	0-0.51
**BAS**	0.01±0.00	0.05±0.01	0.02±0.01	0.05±0.02	0.02±0.01	0.06±0.02	0-0.12
**NEU%**	10.84±5.25	9.66±1.95	15.00±3.37	11.88±0.64	19.30±4.50	14.70±5.62	6.5-50.0
**LYM%**	85.72±5.24	85.38±3.44	81.30±3.64	80.70±1.29	75.40±4.99	78.84±6.38	40.0-92.0
**MON%**	1.58±0.33	2.30±0.71	1.92±0.23	4.14±0.26	2.38±0.47	3.46±1.08	0.9-18.0
**EOS%**	1.42±0.29	2.24±1.10	1.40±0.21	2.66±0.55	2.36±0.78	2.24±0.29	0-7.5
**BAS%**	0.44±0.08	0.42±0.07	0.38±0.13	0.62±0.20	0.56±0.16	0.76±0.27	0-1.5
**RBC**	9.62±0.35	9.17±0.43	9.57±0.23	8.86±0.51	9.07±0.61	8.97±0.43	6.50-11.50
**HGB**	152.20±3.60	148.60±6.41	150.80±5.78	144.80±7.55	142.40±7.66	145.20±4.79	110-165
**HCT**	46.24±1.76	44.30±1.73	46.30±1.36	43.78±2.29	43.52±2.42	43.78±1.39	35.0-55.0
**MCV**	48.06±0.62	48.32±0.54	48.38±0.47	49.46±0.57	48.04±0.86	48.82±1.03	41.0-55.0
**MCH**	15.84±0.22	16.22±0.25	15.74±0.31	16.38±0.40	15.76±0.27	16.24±0.27	13.0-18.0
**MCHC**	329.60±5.46	335.20±2.79	325.40±6.15	331.20±4.49	327.60±2.73	332.40±2.33	300-360

**PLT**	1051.40±129.56	1028.40±373.36	1055.00±118.87	940.00±169.35	1526.40±322.21	1190.80±168.04	400-1600
**MPV**	4.68±0.22	4.82±0.34	4.56±0.05	4.78±0.04	4.58±0.10	4.78±0.07	4.0-6.2
**PDW**	15.18±0.07	15.28±0.07	15.30±0.11	15.22±0.07	15.14±0.12	15.18±0.12	12.0-17.5
**PCT**	0.49±0.07	0.48±0.17	0.48±0.05	0.45±0.08	0.70±0.13	0.57±0.08	0.100-0.780

Plasma samples were obtained 1 week and 2 weeks after treatment. WBC, white blood cell; NEU, neutrophil; LYM, lymphocyte; MON, monocyte; EOS, eosinophilic cell; BAS, basophil; RBC, red blood cell; HGB, hemoglobin; HCT, hematocrit; MCV, mean corpuscular volume; MCH, mean corpuscular hemoglobin; MCHC, mean corpuscular hemoglobin concentration; PLT, Platelets; MPV, mean platelet volume; PDW, platelet distribution width; PCT, thrombocytocrit.

Table S2 Comparison of the relaxivity values of Gd-HFn with the major Gd-based contrast agents reported in the literature.

Contrast agent	Structure	r_1 (mM^{-1} s^{-1})	r_2 (mM^{-1} s^{-1})	Refs
**Magnevist®**	Gd-DTPA	3.9-4.3 (1.5 T)<br>3.5-3.9 (3 T)<br>3.5 ± 0.1 (1.4 T)	3.8-5.4 (1.5 T)<br>4.3-6.1 (3 T)<br>5.1 ± 0.2 (1.4 T)	[1,10]
**Omniscan®**	Gd-DTPA-BMA	4.0-4.6 (1.5T)<br>3.8-4.2 (3T)	4.2-6.2 (1.5 T)<br>4.7-6.5 (3 T)	[2]
**ProHance®**	Gd-DO3A-HP	3.9-4.3 (1.5 T)<br>3.5-3.9 (3 T)	4.2-5.8 (1.5 T)<br>4.8-6.6 (3 T)	[2,9]
**Dotarem®**	Gd-DOTA	3.94 (0.25T)<br>2.96-3.8 (1.5 T)<br>3.3-3.7 (3 T)<br>2.85 (9.4)	3.4-5.2 (1.5 T)<br>4.0-5.8 (3 T)	[3,5,8,9]
**Multihance®**	Gd-BOPTA	6.0-6.6 (1.5 T)<br>5.2-5.8 (3 T)	7.8-9.6 (1.5 T)<br>10.0-12.0 (3 T)	[3]
**OptiMARK®**	Gd-DTPA-BMEA	4.4-5.0 (1.5 T)<br>4.2-4.8 (3 T)	4.3-6.1 (1.5 T)<br>5.0-6.8 (3 T)	[3]
**C-Cha-DOTA**	(Gd^{3+})-chelating 1,4,7,10-tetraazacyclododecane-1,4,7,10-tetraacetic acid (DOTA)	19.5 (sphere),<br>17.2 (fiber)	/	[4]
**Ppdf-Gd**	piX-PEG8-SSSPLGLAK (DOTA)-PEG6-F4	28.2 (sphere),<br>51.5 (fiber) (0.25 T)	/	[5]
**SMNs-Gd, FMNs-Gd**	spherical micellar nanoparticles (Gd^{3+}), fibril-shaped micellar nanoparticles (Gd^{3+})	15.6, 18.5 (0.5 T)	/	[6]
**ultrasmall gadolinium oxide**	poly(acrylic acid-co-maleic acid) (PAAMA) -coated ultrasmall Gd_2O_3	40.6 (3 T)	63.34 (3 T)	[7]
**PEG-P(Lys-DOTA-Gd)**	the micelle-forming poly(ethylene glycol)-b-poly(lysine)	13.31 (1.5 T)<br>5.54 (9.4 T)	/	[8]
**Elucirem® (FDA approved in 2022)**	Gadopiclenol<br>$C_{35}H_{54}GdN_7O_{15}$	12.8 (1.4 T)<br>11.6 (3 T)	/	[9]

**GONP-12**	Gd_2O_3 -PAMPS-LA	63.0±4.4 (9.4 T)	73.5±2.4 (9.4 T)	[10]
**Gadolinium Oxide Nanoparticles**	PASA-coated Gd_2O_3 nanoparticles	19.1 (3 T)	53.7 (3 T)	[11]
**RGD2**	ES-GON5-PAA@RGD2	68.7±2.3 (1.5 T)<br>19.9±0.8 (7 T)	70.5±1.6 (1.5 T)<br>54.8±2.7 (7 T)	[12]
**AFt–C4 NPs**	Gd(III) compound (C4) based on Apoferritin	3.3 (0.5 T)	/	[13]
**Magnetoferritin**	Protein-coated	8 (1.5 T)	218 (1.5 T)	[6]
**The proposed MRI contrast agent**	HFn-Gd	549 (1.5 T)<br>428 (3 T)	1555 (1.5 T)<br>1286 (3 T)	This work

References:

1. Caravan P, Ellison J, McMurry T, Lauffer R. Gadolinium (III) chelates as MRI contrast agents: structure, dynamics, and applications. Chem Rev, 1999; 99(9): 2293-2352.
2. Hao D, Ai T, Goerner F, Hu X, Runge VM, Tweedle M. MRI contrast agents: basic chemistry and safety. J Magn Reso Imaging, 2012; 36(5): 1060-1071.
3. Yan GP, Robinson L, Hogg P. Magnetic resonance imaging contrast agents: overview and perspectives. Radiography, 2007; 13: e5-e19.
4. Kim I, Han EH, Ryu J, Min JY, Ahn H, Chung YH, et al. One-dimensional supramolecular nanoplatforms for theranostics based on co-assembly of peptide amphiphiles. Biomacromolecules, 2016; 17(10): 3234-3243.
5. Zhang J, Mu YL, Ma ZY, Han K, Han HY. Tumor-triggered transformation of chimeric peptide for dual-stage-amplified magnetic resonance imaging and precise photodynamic therapy. Biomaterials, 2018; 182: 269-278.
6. Randolph LM, LeGuyader CL, Hahn ME, Andolina CM, Patterson JP, Mattrey RF, et al. Polymeric Gd-DOTA amphiphiles form spherical and fibril-shaped nanoparticle MRI contrast agents, Chem Sci. 2016; 7(7): 4230-4236.
7. Jang YJ, Liu S, Yue H, Park JA, Cha H, Ho SL, et al. Hydrophilic biocompatible poly (acrylic acid-co-maleic acid) polymer as a surface-coating ligand of ultrasmall Gd2O3 nanoparticles to obtain a high r1 value and T1 MR images. Diagnostics, 2020; 11(1): 2.
8. Yokoyama M, Shiraishi K. Stability evaluation of Gd chelates for macromolecular MRI contrast agents. Magn Reson Mater Phy, Biology and Medicine, 2020; 33: 527-536.
9. Lancelot E, Raynaud JS, Desché P. Current and future MR contrast agents: seeking

a better chemical stability and relaxivity for optimal safety and efficacy. Invest Radiol, 2020; 55(9): 578-588.
10. Stinnett G, Taheri N, Villanova J, Bohloul A, Guo X, Esposito EP, et al. 2D gadolinium oxide nanoplates as T1 magnetic resonance imaging contrast agents. Adv Healthc Mater, 2021; 10(11): 2001780.
11. Marasini S, Yue H, Ghazanfari A, Ho SL, Park JA, Kim S, et al. Polyaspartic acid-coated paramagnetic gadolinium oxide nanoparticles as a dual-modal t1 and t2 magnetic resonance imaging contrast agent. Applied Sciences, 2021; 11(17): 8222.
12. Shen Z, Fan W, Yang Z, Liu Y, Bregadze VI, Mandal SK, et al. Exceedingly small gadolinium oxide nanoparticles with remarkable relaxivities for magnetic resonance imaging of tumors. Small, 2019; 15(41): 1903422.
13. Man X, Yang T, Li W, Li S, Xu G, Zhang Z, et al. Developing a gadolinium (III) compound based on apoferritin for targeted magnetic resonance imaging and dual-modal therapy of cancer. J Med Chem, 2023; 66(11): 7268–7279.
